# Data for temporal facial nerve recovery in Ramsay Hunt syndrome following intratympanic steroid therapy

**DOI:** 10.1016/j.dib.2020.105549

**Published:** 2020-04-23

**Authors:** Akira Inagaki, Toshiya Minakata, Sachiyo Katsumi, Shingo Murakami

**Affiliations:** Departments of Otolaryngology, Head and Neck Surgery, Nagoya City University, Graduate School of Medical Sciences and Medical School, Nagoya, Japan

**Keywords:** Ramsay Hunt syndrome, House-Brackmann grade, Intratympanic steroid therapy, Recovery, Propensity score matching

## Abstract

This article contains data related to the research article entitled “Concurrent treatment with intratympanic dexamethasone improves facial nerve recovery in Ramsay Hunt syndrome” (Akira Inagaki, Toshiya Minakata, Schiyo Katsumi, Shingo Murakami) [1]. This data article reports the protocol for a clinical trial investigating the benefit of intratympanic steroid therapy on facial recovery in Ramsay Hunt syndrome and temporal facial recovery. The data included in this article are as follows: inclusion and exclusion criteria, the treatment protocol of steroids and antiviral therapies, facial recovery as assessed by the House–Brackmann scale in all enrolled patients, House–Brackmann scores in patients with a poor electrophysiological result, and House–Brackmann scores after propensity score matching. This article will be useful for related investigations or clinical practices in the future by serving as a model and benchmark.

Specifications Table

**Table:** 

Subject	Neurology
Specific subject area	Facial palsy
Type of data	Tables
How data were acquired	electronic health record system
Data format	Analysed and filtered
Parameters for data collection	House-Brackmann grade [2]
Description of data collection	The degree of facial nerve dysfunction was assessed independently by facial nerve specialists using the House-Brackmann grading and averaged. To determine the most severe grade, at least two examinations were performed at 4–7-day intervals during the initial 5–17 days after onset; recovery was monitored by examinations performed at 1 month (days 25–35), 3 months (days 75–105), 6 months (days 167–197), 9 months (days 258–288), and 12 months (days 350–380).
Data source location	Nagoya, Japan
Data accessibility	With the article
Related research article	Akira Inagaki, Toshiya Minakata, Schiyo Katsumi, Shingo Murakami, Concurrent treatment with intratympanic dexamethasone improves facial nerve recovery in Ramsay Hunt syndrome, Journal of Neurological Sciences, in Press

## Value of the Data

• The dataset included in this article will be useful for further studies investigating treatment outcomes in patients with Ramsay Hunt syndrome presenting with moderately severe to severe facial palsy.
• The dataset of relatively rare conditions will benefit healthcare professionals and medical researchers who should find these data useful for comparative purposes.
• The dataset can be used as benchmark data of facial recovery in Ramsay Hunt syndrome with moderately severe to severe facial palsy for future studies on new therapies and of other aetiologies presenting with facial palsy.

## 1 Data Description

This article contains data related to the research article entitled “Concurrent treatment with intratympanic dexamethasone improves facial nerve recovery in Ramsay Hunt syndrome” (Akira Inagaki, Toshiya Minakata, Schiyo Katsumi, Shingo Murakami) [1]. The first part of the article presents the detailed protocol of the clinical trial. Table 1 summarizes the inclusion and exclusion criteria of this trial. Table 2 summarizes standard oral steroid and antiviral therapies and the concurrent ITST dexamethasone dose that were used as the intervention. Patients who received systemic steroids and antiviral treatment at the standard dose or higher were recruited as historical controls. The second part of the article shows facial recovery datasets in patients with moderately severe to severe Ramsay Hunt syndrome in the historical controls and patients with concurrent Intratympanic steroid therapy (ITST) as indicated by the House–Brackmann grade. Table 3 shows recovery from facial palsy in all enrolled patients in the two groups. Table 4 presents recovery from facial palsy in patients in the two groups with a poor electrophysiological result. Table 5 summarizes the baseline characteristics and outcomes in patients in the two groups after propensity score adjustment. Table 6 shows recovery from facial palsy in patients in the two groups after propensity score adjustment. All the raw datasets in each table are included in the supplementary data.

**Table 1:** Inclusion and exclusion criteria

Inclusion criteria
1. Diagnosis of Ramsay Hunt syndrome
2. Protocol able to be initiated within 7 days of onset
3. Age 20 years or older
4. Severe facial nerve palsy (House-Brackmann grade IV or higher)
Exclusion criteria
1. Nonviral inflammation in the middle ear
2. Bacterial disease at carriage state
3. Middle ear or inner ear anomaly preventing injection into middle ear
4. Glycated hemoglobin >6.5%
5. Blood urea nitrogen level >25 mg/dl or serum creatinine level >2.0 mg/dl
6. Alanine transaminase > 100 U/l or aspartate transaminase >100 U/l
7. Severely protracted wound healing
8. Signs of central facial nerve palsy or other neurological disease that could potentially affect facial function
9. Pregnancy or possible pregnancy
10. Participation in another clinical trial within the past 3 months
11. Recurrent facial palsy
12. For patients receiving concurrent intratympanic steroid therapy: pretreatment other than 60 mg of prednisolone in combination with 3,000 mg oral valaciclovir within 2 days. For control patients: treatment with prednisolone and/or valacyclovir at dosages lower than those in Table 1.
14. Other considerations that would make a patient inappropriate for the clinical trial.

**Table 2:** Standard oral steroid and antiviral therapies and concurrent ITST dexamethasone dose

Day	Prednisolone dose (mg)	Valaciclovir dose (mg)	Concurrent ITST dexamethasone dose (mg)
1	60	3000	1.65
2	60	3000	1.65
3	60	3000	1.65
4	60	3000	1.65
5	60	3000	1.65
6	30	3000	1.65
7	30	3000	1.65
8	30		1.65
9	10		1.65
10	10		1.65

Doses of prednisolone and valacyclovir are those specified in the standard protocols for the treatment of severe Ramsay Hunt syndrome. All the patients enrolled in this dataset started on intratympanic steroid therapy on day 1.

**Table 3:** Outcomes of concurrent treatment with ITST and systemic steroids in all enrolled patients: Recovery from facial palsy as indicated by House-Brackmann grade

	HB grade in all enrolled patients	
	Concurrent ITST/control	Difference/*P*-value
Onset	5.00 ± 0.17 5.20 ± 0.10	0.20/ *P* =0.313
1 month	2.75 ± 0.37/ 3.97 ± 0.28	1.22/ *P* = 0.021*
3 months	1.75 ± 0.35/ 2.77 ± 0.30	1.02/ *P* = 0.091
6 months	1.25 ± 0.18/ 2.21 ± 0.25	0.96/ *P* = 0.034*
9 months	1.08 ± 0.08/ 1.91 ± 0.19	0.83/ *P* =0.011*
12 months	1.08 ± 0.08/ 1.88 ± 0.19	0.80/ *P* = 0.011*

*P < 0.05; **P < 0.01.

**Table 4:** Outcomes of concurrent treatment with ITST and systemic steroids in patients with a poor electrophysiological result: Recovery from facial palsy as indicated by House-Brackmann grade

	HB grade in patients with a poor prognostic factor	
	Concurrent ITST/control	Difference/*P*-value
Onset	5.20 ± 0.20 / 5.56 ± 0.18	0.36 / P =0.228
1 month	4.00 ± 0.32 / 5.33 ± 0.24	1.33 / P = 0.003**
3 months	2.60 ± 0.68 / 4.67 ± 0.41	2.07 / P =0.008**
6 months	1.60 ± 0.40 / 3.56 ± 0.41	1.96 / P = 0.005**
9 months	1.20 ± 0.20 / 2.89 ± 0.35	1.69 / P = 0.006**
12 months	1.20 ± 0.20 / 2.78 ± 0.36	1.58 / P =0.005**

*P < 0.05; **P < 0.01.

**Table 5:** Baseline characteristics and outcomes in patients with moderate-severe to severe Ramsay Hunt syndrome in the two groups after propensity score adjustment

Variable	Group		
	ITST (*n*=12) (range)	Systemic steroid (*n*=24) (range)	*P*-value
Age (years)	38.2 ± 4.2 (21–74)	46.5 ± 3.4 (24–72)	0.261
Time since onset of first systemic steroid treatment (days)	3.16 ± 0.55 (1–7)	1.88 ± 0.33 (0–7)	0.041*
Most severe HB grade	5.00 ± 0.17	5.08 ± 0.12	0.702
HB grade at 12 months	1.25 ± 0.16	1.92 ± 0.23	0.013*
Total amount of systemic prednisolone (mg)	410 (fixed) Dex 16.5 mg	566 ± 31 (420–1080)	0.105
Recovery to HB grade I	11/12 (93%)	12/24 (50%)	0.005^†^ **
Recovery to HB grade I Adjusted odds ratio (95% CI)*	26.1 (1.09–628.37)		0.044

Dex; dexamethasone; HB, House-Brackmann *P < 0.05; **P < 0.01.

**Table 6:** Recovery from facial palsy in patients with moderate-severe to severe Ramsay Hunt syndrome in the two groups after propensity score

	Propensity score-adjusted HB grades	
	Concurrent ITST (*n*=12) /control (*n*=12)	Difference/*P*-value
Onset (most severe grade)	5.00 ± 0.17/5.08 ± 0.12	0.08/0.702
1 month	2.75 ± 0.37/3.92 ± 0.34	1.17/0.034*
3 months	1.75 ± 0.35/2.75 ± 0.34	1.00/0.104
6 months	1.25 ± 0.18/2.21 ± 0.30	0.96/0.04*
9 months	1.08 ± 0.08/1.92 ± 0.23	0.84/0.013*
12 months	1.08 ± 0.08/1.92 ± 0.23	0.84/0.013*

*P<0.05.

## 2 Experimental Design, Materials, and Methods

### 2.1 Trial Design

This prospective, open-label trial was performed at a single institution in Japan and included a historical control group. The primary outcomes were the rate and extent of recovery of facial nerve function as assessed by the House-Brackmann (HB) grading system [2] at 1, 3, 6, 9, and 12 months after onset of facial palsy.

### 2.2 Participants and Setting

Participants were patients aged ≥20 years who received treatment for Ramsay Hunt syndrome in the otolaryngology clinic at Nagoya City University Hospital, Nagoya, Japan between March 2014 and December 2015 (concurrent ITST group) or between January 2007 and February 2014 (historical control group). The protocol was approved by the Institutional Review Board of Nagoya City University (Clinical Trial Registration Number, 41-13-0004; UMIN trial registration: umin.ac.jp; UMIN000031107). All patients who were considered candidates for concurrent ITST were informed about the protocol, invited to participate, and enrolled after written informed consent was obtained. Patients in the historical control group were informed about the protocol and given the opportunity to opt out. The need for permission to review medical records retrospectively was waived (approval number 60-18-0001).

Ramsay Hunt syndrome was diagnosed as facial palsy with an associated erythematous vesicular rash of the pinna or mouth [3]. Inclusion and exclusion criteria are summarized in Table 1. ITST was performed for 10 consecutive days, concurrent with a fixed dose of systemic prednisolone (starting at 60 mg/day and tapered thereafter; total dose, 410 mg) and valacyclovir 3000 mg/day for 7 days, starting on the day of enrolment (Table 2). Patients identified in the medical records to have Ramsay Hunt syndrome and to have received systemic steroids and antiviral treatment at the standard dose or higher were retrospectively recruited as historical controls.

For administration of ITST, patients were instructed to remain supine with the head tilted 40–45° to the unaffected side and bent slightly backwards. After administering local aesthesia, we introduced a small ventilation hole into the tympanic membrane and injected 0.5 ml of dexamethasone (3.3 mg/ml) into the tympanic cavity using a 23-G spinal needle. The patients were asked to avoid moving the head, swallowing, or yawning for 15 min after the injection [4].

### 2.3 Patient Evaluation

In both groups, the degree of facial nerve dysfunction was assessed independently by facial nerve specialists using the 6-point HB grading system (grade I, normal; VI, total facial paralysis) [2] and averaged. To determine the most severe grade, at least two examinations were performed at 4–7-day intervals during the initial 5–17 days after onset; recovery was monitored by examinations performed at 1 month (days 25–35), 3 months (days 75–105), 6 months (days 167–197), 9 months (days 258–288), and 12 months (days 350–380).

Electroneurography (ENoG) were performed between 7 and 12 days after onset of facial palsy using the MEB-2300-Neuropack X1 Measuring System (Nihon Kohden, Tokyo, Japan).

### 2.4 Propensity score matching

Propensity score estimates representing the probability of reaching House-Brackmann (HB) grade I at 12 months were generated separately for the concurrent ITST group and the control group using JMP software (version 13.2.1; SAS Institute, Cary, NC). A logistic regression model that incorporated the variables used to generate the backward stepwise logistic regression model for multivariate analysis (age, sex, days from onset to first systemic steroid treatment, and initial HB grade, see Subjects And Methods in Statistics for details) was created. Following propensity score matching, patients with Ramsay Hunt syndrome were matched within each group by the 2:1 nearest neighbor (Greedy-type) matching function of the R package MatchIt [5] (R Foundation for Statistical Computing, Vienna, Austria) with a caliper width of a 0.2 standard deviation of the propensity score logit because of the small sample size in the ITST group. Matching was performed without replacement, and nonmatched results were discarded. Improvement in covariate balance following matching was measured using logistic regression conditioned on the specific pair identification assigned to each match. Covariates and outcome incidences were compared between each group by the Student's t-test for continuous variables and Fisher's exact test for categorical variables using Sigmaplot software (Wavemetrics, Lake Oswego, OR).

A backward stepwise logistic regression model was used to identify a multivariate model appropriate for deriving an adjusted odds ratio. Nonsignificant (P>0.1) independent variables were eliminated through a stepwise backward process to produce the most parsimonious final regression model (as judged by the Akaike information criterion; JMP version 13.2.1). Based on the backward stepwise method, the most severe HB grade, age, and sex (P =0.0004, 0.009 and 0.053) were left in the final model.

## Appendix Supplementary materials

Image, application 1
