# Supplementary material for: Data for temporal facial nerve recovery in Ramsay Hunt syndrome following intratympanic steroid therapy
Source: Data Brief. 2020 Apr 23;30:105549. doi: 10.1016/j.dib.2020.105549 (PMC7184121; doi:10.1016/j.dib.2020.105549)
Supplement: Supplementary file 1 [file mmc1.docx]

**Supplementary Data**

**Data for temporal facial nerve recovery in Ramsay Hunt syndrome following intratympanic steroid therapy**

**Authors**

Akira Inagaki^1^, Toshiya Minakata^1^, Sachiyo Katsum^1^i, Shingo Murakami^1^

**Affiliations**

1. Departments of Otolaryngology, Head and Neck Surgery, Nagoya City University

Graduate School of Medical Sciences and Medical School, Nagoya, Japan

**Supplementary Data**

**Supplementary Data 1.** Raw data set for Table 3.

**Supplementary Data 2.** Raw data set for Table 4.

**Supplementary Data 3.** Raw data set for Table 5.

**Supplementary Data 4.** Raw data set for Table 6.

**Supplementary Data 1.** Raw data set for Table 3, Outcomes of concurrent treatment with ITST and systemic steroids in all enrolled patients: Recovery from facial palsy as indicated by House-Brackmann grade.

A. Concurrent ITST group

| Patient No | House-Brackmann Grade/Concurrent ITST (n=6) | | | | | |
| --- | --- | --- | --- | --- | --- | --- |
|  | Worst grade | 1Mo | 3Mo | 6Mo | 9Mo | 12Mo |
| 1 | 5 | 4 | 3 | 2 | 1 | 1 |
| 2 | 5 | 3 | 2 | 1 | 1 | 1 |
| 3 | 6 | 5 | 5 | 3 | 2 | 2 |
| 4 | 5 | 4 | 2 | 1 | 1 | 1 |
| 5 | 5 | 4 | 1 | 1 | 1 | 1 |
| 6 | 5 | 3 | 1 | 1 | 1 | 1 |
| 7 | 4 | 1 | 1 | 1 | 1 | 1 |
| 8 | 5 | 2 | 1 | 1 | 1 | 1 |
| 9 | 5 | 2 | 2 | 1 | 1 | 1 |
| 10 | 4 | 1 | 1 | 1 | 1 | 1 |
| 11 | 6 | 2 | 1 | 1 | 1 | 1 |
| 12 | 5 | 2 | 1 | 1 | 1 | 1 |

ITST, intratympanic steroid therapy.

B. Control group

| Patient No | House-Brackmann Grade/Control (n=9) | | | | | |
| --- | --- | --- | --- | --- | --- | --- |
|  | Worst grade | 1Mo | 3Mo | 6Mo | 9Mo | 12Mo |
| 1 | 5 | 5 | 5 | 3 | 2 | 2 |
| 2 | 5 | 4 | 3 | 2 | 2 | 2 |
| 3 | 5 | 5 | 3 | 2 | 2 | 2 |
| 4 | 6 | 5 | 4 | 3 | 3 | 3 |
| 5 | 6 | 6 | 6 | 5 | 5 | 5 |
| 6 | 6 | 6 | 5 | 5 | 3 | 3 |
| 7 | 5 | 5 | 4 | 3 | 2 | 2 |
| 8 | 6 | 6 | 6 | 5 | 3 | 2 |
| 9 | 6 | 6 | 6 | 4 | 4 | 4 |
| 10 | 5 | 5 | 4 | 3 | 3 | 3 |
| 11 | 5 | 3 | 1 | 1 | 1 | 1 |
| 12 | 6 | 3 | 1 | 1 | 1 | 1 |
| 13 | 6 | 6 | 5 | 5 | 4 | 4 |
| 14 | 6 | 3 | 1 | 1 | 1 | 1 |
| 15 | 4 | 4 | 1 | 1 | 1 | 1 |
| 16 | 5 | 5 | 4 | 4 | 3 | 3 |
| 17 | 5 | 2 | 1 | 1 | 1 | 1 |
| 18 | 5 | 3 | 2 | 1 | 1 | 1 |
| 19 | 5 | 3 | 2 | 1 | 1 | 1 |
| 20 | 5 | 1 | 1 | 1 | 1 | 1 |
| 21 | 5 | 1 | 1 | 1 | 1 | 1 |
| 22 | 6 | 6 | 4 | 4 | 3 | 3 |
| 23 | 5 | 5 | 1 | 1 | 1 | 1 |
| 24 | 5 | 5 | 4 | 3 | 3 | 3 |
| 25 | 5 | 3 | 2 | 2 | 2 | 2 |
| 26 | 5 | 4 | 3 | 1 | 1 | 1 |
| 27 | 6 | 5 | 3 | 2 | 2 | 2 |
| 28 | 4 | 1 | 1 | 1 | 1 | 1 |
| 29 | 4 | 1 | 1 | 1 | 1 | 1 |
| 30 | 5 | 4 | 1 | 1 | 1 | 1 |
| 31 | 5 | 3 | 1 | 1 | 1 | 1 |
| 32 | 5 | 5 | 4 | 3 | 2 | 2 |
| 33 | 5 | 2 | 1 | 1 | 1 | 1 |
| 34 | 5 | 4 | 2 | 1 | 1 | 1 |

**Supplementary Data 2.** Raw data set for Table 4, Outcomes of concurrent treatment with ITST and systemic steroids in patients with a poor electrophysiological result: Recovery from facial palsy as indicated by House-Brackmann grade

A. Concurrent ITST group

| Patient No | House-Brackmann Grade/Concurrent ITST (n=6) | | | | | |
| --- | --- | --- | --- | --- | --- | --- |
|  | Worst grade | 1Mo | 3Mo | 6Mo | 9Mo | 12Mo |
| 1 | 6 | 5 | 4 | 2 | 2 | 1 |
| 2 | 5 | 4 | 3 | 2 | 1 | 1 |
| 3 | 5 | 3 | 2 | 1 | 1 | 1 |
| 4 | 6 | 5 | 5 | 3 | 2 | 2 |
| 5 | 5 | 4 | 2 | 1 | 1 | 1 |
| 9 | 5 | 4 | 1 | 1 | 1 | 1 |

ITST, intratympanic steroid therapy.

B. Control group

| Patient No | House-Brackmann Grade/Control (n=9) | | | | | |
| --- | --- | --- | --- | --- | --- | --- |
|  | Worst grade | 1Mo | 3Mo | 6Mo | 9Mo | 12Mo |
| 1 | 5 | 5 | 5 | 3 | 2 | 2 |
| 2 | 5 | 4 | 3 | 2 | 2 | 2 |
| 3 | 5 | 5 | 3 | 2 | 2 | 2 |
| 4 | 6 | 5 | 4 | 3 | 3 | 3 |
| 5 | 6 | 6 | 6 | 5 | 5 | 5 |
| 6 | 6 | 6 | 5 | 5 | 3 | 3 |
| 7 | 5 | 5 | 4 | 3 | 2 | 2 |
| 8 | 6 | 6 | 6 | 5 | 3 | 2 |
| 9 | 6 | 6 | 6 | 4 | 4 | 4 |

**Supplementary Data 3.** Raw data set for Table 5, Baseline characteristics and outcomes in patients with moderate-severe to severe Ramsay Hunt syndrome in the two groups after propensity score adjustment

1. Age

| Age (year) | |
| --- | --- |
| Concurrent ITST after propensity score matching (n=12) | Control after propensity score matching (n=24) |
| 21 | 24 |
| 23 | 26 |
| 26 | 28 |
| 31 | 28 |
| 32 | 30 |
| 34 | 30 |
| 34 | 31 |
| 41 | 32 |
| 45 | 36 |
| 48 | 38 |
| 49 | 40 |
| 74 | 42 |
|  | 44 |
|  | 48 |
|  | 51 |
|  | 55 |
|  | 58 |
|  | 63 |
|  | 66 |
|  | 67 |
|  | 68 |
|  | 68 |
|  | 72 |
|  | 72 |

B. Time since onset of first systemic steroid treatment (days)

| Time since onset of first systemic steroid treatment (days) | |
| --- | --- |
| Concurrent ITST after propensity score matching (n=12) | Control after propensity score matching (n=24) |
| 1 | 0 |
| 1 | 0 |
| 1 | 0 |
| 2 | 0 |
| 2 | 1 |
| 3 | 1 |
| 3 | 1 |
| 4 | 1 |
| 4 | 1 |
| 5 | 1 |
| 5 | 1 |
| 7 | 2 |
|  | 2 |
|  | 2 |
|  | 2 |
|  | 2 |
|  | 2 |
|  | 2 |
|  | 3 |
|  | 3 |
|  | 3 |
|  | 4 |
|  | 4 |
|  | 7 |

C. Total amount of systemic prednisolone (mg)

| Total amount of systemic prednisolone (mg)* | |
| --- | --- |
| Concurrent ITST after propensity score matching (n=12) | Control after propensity score matching (n=24) |
| 480 | 410 |
| 480 | 410 |
| 480 | 410 |
| 480 | 410 |
| 480 | 410 |
| 480 | 420 |
| 480 | 450 |
| 480 | 500 |
| 480 | 500 |
| 480 | 525 |
| 480 | 525 |
| 480 | 585 |
|  | 585 |
|  | 585 |
|  | 585 |
|  | 585 |
|  | 585 |
|  | 585 |
|  | 640 |
|  | 652 |
|  | 660 |
|  | 670 |
|  | 760 |
|  | 1080 |

* Equivalent dosage of 16.5mg of dexamethasone is postulated as 70 mg of prednisolone. ITST, intratympanic steroid therapy.

**Supplementary Data 4.** Raw data set for Table 6, Recovery from facial palsy in patients with moderate-severe to severe Ramsay Hunt syndrome in the two groups after propensity score

A. Concurrent ITST group

| Patient No | House-Brackmann Grade/Concurrent ITST after propensity score matching (n=12) | | | | | |
| --- | --- | --- | --- | --- | --- | --- |
|  | Worst grade | 1Mo | 3Mo | 6Mo | 9Mo | 12Mo |
| 1 | 5 | 4 | 3 | 2 | 1 | 1 |
| 2 | 5 | 3 | 2 | 1 | 1 | 1 |
| 3 | 6 | 5 | 5 | 3 | 2 | 2 |
| 4 | 5 | 4 | 2 | 1 | 1 | 1 |
| 5 | 5 | 4 | 1 | 1 | 1 | 1 |
| 6 | 5 | 3 | 1 | 1 | 1 | 1 |
| 7 | 4 | 1 | 1 | 1 | 1 | 1 |
| 8 | 5 | 2 | 1 | 1 | 1 | 1 |
| 9 | 5 | 2 | 2 | 1 | 1 | 1 |
| 10 | 4 | 1 | 1 | 1 | 1 | 1 |
| 11 | 6 | 2 | 1 | 1 | 1 | 1 |
| 12 | 5 | 2 | 1 | 1 | 1 | 1 |

ITST, intratympanic steroid therapy.

B. Control group

| Patient No | House-Brackmann Grade/Control after propensity score matching (n=24) | | | | | |
| --- | --- | --- | --- | --- | --- | --- |
|  | Worst grade | 1Mo | 3Mo | 6Mo | 9Mo | 12Mo |
| 1 | 5 | 5 | 5 | 3 | 2 | 2 |
| 2 | 6 | 5 | 4 | 3 | 3 | 3 |
| 3 | 6 | 6 | 6 | 5 | 5 | 5 |
| 4 | 6 | 6 | 5 | 5 | 3 | 3 |
| 5 | 5 | 5 | 4 | 3 | 2 | 2 |
| 6 | 5 | 5 | 4 | 3 | 3 | 3 |
| 7 | 6 | 6 | 5 | 5 | 4 | 4 |
| 8 | 4 | 4 | 1 | 1 | 1 | 1 |
| 9 | 5 | 5 | 4 | 4 | 3 | 3 |
| 10 | 5 | 2 | 1 | 1 | 1 | 1 |
| 11 | 5 | 3 | 2 | 1 | 1 | 1 |
| 12 | 5 | 1 | 1 | 1 | 1 | 1 |
| 13 | 5 | 1 | 1 | 1 | 1 | 1 |
| 14 | 5 | 5 | 1 | 1 | 1 | 1 |
| 15 | 5 | 5 | 4 | 3 | 3 | 3 |
| 16 | 5 | 3 | 2 | 2 | 2 | 2 |
| 17 | 5 | 4 | 3 | 1 | 1 | 1 |
| 18 | 6 | 5 | 3 | 2 | 2 | 2 |
| 19 | 4 | 1 | 1 | 1 | 1 | 1 |
| 20 | 4 | 1 | 1 | 1 | 1 | 1 |
| 21 | 5 | 4 | 1 | 1 | 1 | 1 |
| 22 | 5 | 3 | 1 | 1 | 1 | 1 |
| 23 | 5 | 5 | 4 | 3 | 2 | 2 |
| 24 | 5 | 4 | 2 | 1 | 1 | 1 |
